# Distribution of segmental duplications in the context of higher order chromatin organisation of human chromosome 7

**DOI:** 10.1186/1471-2164-15-537

**Published:** 2014-06-29

**Authors:** Grit Ebert, Anne Steininger, Robert Weißmann, Vivien Boldt, Allan Lind-Thomsen, Jana Grune, Stefan Badelt, Melanie Heßler, Matthias Peiser, Manuel Hitzler, Lars R Jensen, Ines Müller, Hao Hu, Peter F Arndt, Andreas W Kuss, Katrin Tebel, Reinhard Ullmann

**Affiliations:** Max Planck Institute for Molecular Genetics, Ihnestraße 63-73, 14195 Berlin, Germany; Department of Biology, Chemistry and Pharmacy, Free University Berlin, 14195 Berlin, Germany; Department of Human Genetics, University Medicine Greifswald, and Interfaculty Institute of Genetics and Functional Genomics, University of Greifswald, Fleischmannstraße 42-44, 17475 Greifswald, Germany; Wilhelm Johannsen Centre for Functional Genome Research, Department of Cellular and Molecular Medicine, University of Copenhagen, Blegdamsvej 3, DK-2200 Copenhagen, Denmark; Institute for Theoretical Chemistry, University of Vienna, Waehringer Straße 17, A-1090 Vienna, Austria; Unit Experimental Research, Department of Product Safety, Federal Institute for Bundeswehr Institute of Radiobiology affiliated, the University of Ulm, Neuherbergstraße 11, 80937 Munich, Germany

**Keywords:** Higher order chromatin organisation, Segmental duplication, Williams-Beuren syndrome, Chromosome evolution, Hi-C

## Abstract

**Background:**

Segmental duplications (SDs) are not evenly distributed along chromosomes. The reasons for this biased susceptibility to SD insertion are poorly understood. Accumulation of SDs is associated with increased genomic instability, which can lead to structural variants and genomic disorders such as the Williams-Beuren syndrome. Despite these adverse effects, SDs have become fixed in the human genome. Focusing on chromosome 7, which is particularly rich in interstitial SDs, we have investigated the distribution of SDs in the context of evolution and the three dimensional organisation of the chromosome in order to gain insights into the mutual relationship of SDs and chromatin topology.

**Results:**

Intrachromosomal SDs preferentially accumulate in those segments of chromosome 7 that are homologous to marmoset chromosome 2. Although this formerly compact segment has been re-distributed to three different sites during primate evolution, we can show by means of public data on long distance chromatin interactions that these three intervals, and consequently the paralogous SDs mapping to them, have retained their spatial proximity in the nucleus. Focusing on SD clusters implicated in the aetiology of the Williams-Beuren syndrome locus we demonstrate by cross-species comparison that these SDs have inserted at the borders of a topological domain and that they flank regions with distinct DNA conformation.

**Conclusions:**

Our study suggests a link of nuclear architecture and the propagation of SDs across chromosome 7, either by promoting regional SD insertion or by contributing to the establishment of higher order chromatin organisation themselves. The latter could compensate for the high risk of structural rearrangements and thus may have contributed to their evolutionary fixation in the human genome.

**Electronic supplementary material:**

The online version of this article (doi:10.1186/1471-2164-15-537) contains supplementary material, which is available to authorized users.

## Background

Segmental duplications (SDs) are DNA sequences larger than 1 kb, which can be found at least twice with more than 90% sequence similarity in the genome. They are a feature of various eukaryotic genomes, however, they have particularly accumulated during primate evolution [1–3]. Thus the percentage of SDs has increased from about 2% in the New World monkey marmoset (*Callithrix jacchus*) genome [4] to approximately 5% in the human genome [5]. It is not clear what has triggered this recent burst of SDs, but the simultaneous decrease of point mutations and retrotransposition rate argues against that this is owed to a general increase of mutability [2]. Although SDs pose a serious threat to genomic integrity by promoting non-allelic homologous recombination (NAHR), this specific type of DNA copy number variant has been fixed in the genome. One reason for the manifestation of SDs could be their preferential location in gene-rich genomic segments and their high gene content [6, 7]. Several of the duplicated exons appear to be subject of accelerated evolution [8, 9], which has led to neofunctionalisation and subfunctionalisation of duplicated genes [10–14]. However, in most cases mutations have resulted in pseudogenisation of duplicated genes [4, 15, 16], that nevertheless can show remarkably high transcriptional activity [4, 17]. Yet, the large fraction of pericentromeric SDs, which is less gene-rich [18], points at alternative factors that could support positive selection of SDs. For example, SD insertion could also impact gene expression by demarcating euchromatin from transcriptional inactive heterochromatin [19, 20]. Moreover, it has been discussed that SDs, which frequently map to synteny breaks [21–25], may have mediated evolutionary rearrangements that have led to reproductive isolation of their carriers [26]. However, the temporal order of events argues against the impact of SDs on the generation of evolutionary rearrangements in many cases [27, 28]. On the contrary, a recent study supports the idea that the accumulation of SDs may also be the consequence of evolutionary rearrangements rather than their cause [20].

SDs are not evenly distributed across the genome. Instead there are profound differences within and among chromosomes [29, 30]. Apart from large SD clusters in the subtelomeric and pericentromeric regions of most chromosomes, SDs can also accumulate in interstitial hubs [4, 18]. These hubs are characterised by an increased genomic instability, which manifests itself in a high probability of further SD insertion in their flanking regions, a phenomenon termed SD shadowing [31]. Furthermore, such hubs favour the presence of numerous structural variants with many of them having pathological relevance [32]. Yet, it is still uncertain what mechanisms have driven SD aggregation in the first place [33] and whether the *pro rata* contribution of any such mechanism remained the same throughout evolution [34]. A pivotal first step preceding formation of SD hubs may have been the insertion of core SDs [29]. Recombination between repetitive elements may play a role too, as nearly 27% of all SDs are flanked by Alu repeats [35]. In addition, the association of SDs with G4 motifs and other sequence features promoting non-B DNA conformations [19] points at the possible relevance of chromatin conformation for SD insertion.

However, studies investigating SD distribution across the genome have so far based their analysis on the linear genome and have not taken into account its complex three dimensional organisation. Therefore, in this study we combined publicly available data on the three-dimensional organisation of the nucleus [36] with own experimental data in order to explore the distribution of SDs in relation to higher order chromatin organisation. Focusing on chromosome 7 with its particular high content of intrachromosomal and interstitial SDs [7, 22, 37], we demonstrate that paralogous SDs, that have been separated in the course of evolution, are still in close spatial proximity. Proceeding on this observation we have explored a possible role of SDs in sequence directed chromatin organisation and discuss how this may impact the emergence of genomic disorders such as the Williams-Beuren syndrome (WBS).

## Results

### Filtering and bundling of Hi-C interaction bins

We have inferred spatial proximities of intrachromosomal SDs from normalised Hi-C data for chromosome 7 [36] at a resolution of 20 kb. Hi-C is a derivative of the chromosome conformation capture protocol (3C) [38, 39] and facilitates the genome-wide analysis of chromatin interactions within the nucleus. It is a proximity ligation based technology, where DNA is cut, re-ligated and the products are analysed by paired-end sequencing. The frequency of two DNA sequences co-occurring in the same paired-end reads reflects their contact probability within the nucleus across a large population of heterogeneous cells in all phases of the cell cycle.

**Figure 1:**
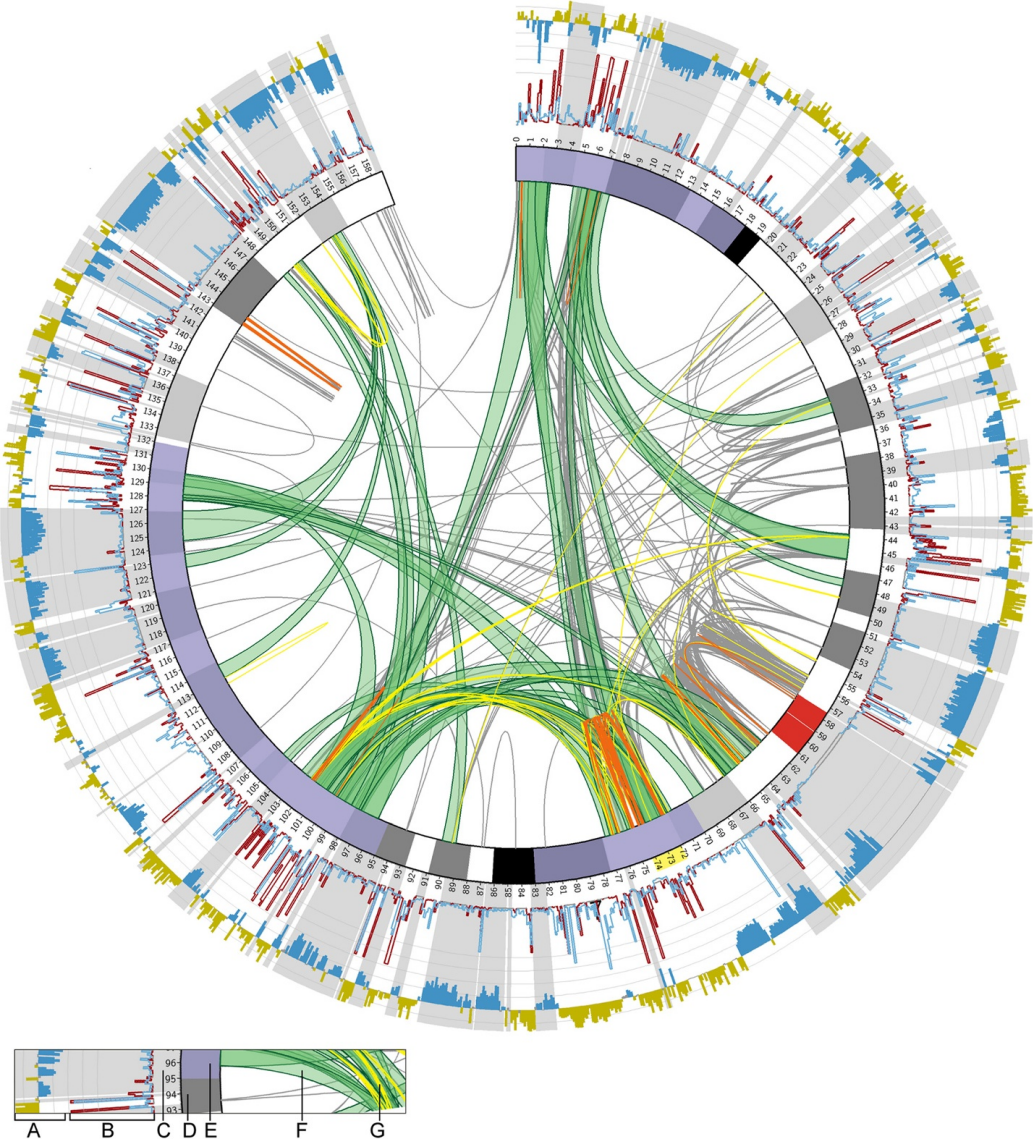
**Distribution of segmental duplications (SDs) and bundled long distance interactions in relation to acetylation of H4K8, transcriptional activity and lamina associated domains on human chromosome 7 (derived from IMR90 unless indicated otherwise). A)** H4K8 acetylation profile, dark yellow: hyperacetylation of H4K8; blue: hypoacetylation of H4K8. **B)** the red and blue curve represent RNA-seq read counts/100 kb bin for coding and non-coding RNA, respectively (IMR91L). **C)** grey areas underlying the two histograms mark lamina associated domains (LADs, Tig3 cells). **D)** idiogram of chromosome 7, the Williams-Beuren syndrome region is highlighted in yellow beside the idiogram (at 72-74 Mb, hg18). **E)** transparent blue shading of the idiogram illustrates the inversion-affected segments of chromosome 7 depicted in Figure 2A-C. Bundled long distance interactions **(F)** and segmental duplications **(G)** are depicted in the inner circle; green ribbons: long distance interactions between genomic regions; grey: SDs with sequence similarity <98%; yellow: SDs with sequence similarity 98-99%; orange: SDs with sequence similarity >99%.

In order to concentrate on the most prevailing Hi-C interactions and to minimise the influence of random noise, we have applied different criteria to filter Hi-C data bins by changing 1) the normalised number of reads necessary to confirm the interaction of two given bins and 2) the minimal genomic distance of interacting bins. For each of these data sets adjacent interaction bins were merged to regions of interaction bundles if their start and target sites locate within an interval of 500 kb, respectively, using Circos tools [40]. Bundling all long distance interactions that have been confirmed by at least 15 interaction counts (=normalised number of paired-end reads) with a minimum interaction span size of at least 25 Mb using the bundling criteria “at least five interaction bins mapping within 500 kb at the start and the target site” to interaction bins, resulted in 33 bundles covering 37.2 Mb in total (i.e. 23.4% of chromosome 7, Additional file 1). In line with the literature, these long distance interaction bundles preferentially connect regions with high transcriptional activity and open chromatin [36, 39, 41] as demonstrated by our RNA-seq and H4K8ac data (Figure 1 and Additional file 2).

In accordance with the preferential insertion of SDs into the gene-rich euchromatic portion of the genome, SD regions have a higher probability to be located within long distance interaction bundles (for chr7: adjusted *p*-value = 1.3332 × 10^−4^, for all chromosomes: adjusted *p*-value = 1.3332 × 10^−4^, 10000 simulations; Additional file 3). In two out of 1474 instances start and target site of long distance interaction bins directly coincide with the location of two SD paralogs (Additional file 2). Although the initial sequence alignment of Hi-C reads, as performed by Dixon et al. [36], employed a mapping quality score chosen to accept unique reads only, there is an apparent risk that some of these long distance interactions are owed to erroneous sequence alignment. Thus, we added a third filter for the Hi-C data bins, namely 3) the exclusion of genomic bins overlapping with SDs. We tested the consequences on the bundling pattern after removing all interacting bins that connect two given SD paralogs (termed IA bins w/o SD paralogs in Additional file 4), as well as ignoring all interaction bins that overlap with any SD at all (termed IA bins w/o any SD in Additional file 4). These filter options are aimed at excluding all short distance interactions that have been misinterpreted as long distance interactions due to false alignment of one side of a paired-end read. While this reduced the number of interaction bins by 0.01% and 9.75% (and 0.14% and 59.77% when only considering long distance interactions; see Additional file 1), interactions of bins adjacent to the removed ones were sufficient to retain the basic triangular interaction pattern (Additional file 4C-F and H). In addition to the filtering of SD overlapping interaction bins at the resolution of 20 kb, we performed a filtering also at the level of paired-end reads starting from the raw Hi-C data [36]. After exclusion of 369559 intrachromosomal paired-end reads that ambiguously mapped to chromosome 7 (affecting 5.11% of intrachromosomal 20 kb interaction bins), data were normalised and bundled (Additional files 1 and 4J).

In order to avoid threshold-induced interpretation bias we have tested in total 12 different combinations of cut-offs and filter criteria (Additionals file 1 and 4) with variations in interaction counts per bin, interaction distance and handling of genomic bins overlapping with known SDs for the bundling of Hi-C data. The intersection of these 12 data sets revealed a core pattern of interactions independent of the threshold used (Additionals files 4H and 5). Therefore it is unlikely that the observed proximities of paralogous SDs are solely result of ambiguous sequence alignments within segmental duplications. However, we want to emphasise that given the paucity of reliable interaction counts within SDs, this statement heavily depends on the interaction patterns of adjacent bins that lack any SDs and is supported by shared regions of interactions as indicated by triangular interaction patterns.

### Chromosomal regions separated in the course of evolution retain spatial proximity

**Figure 2:**
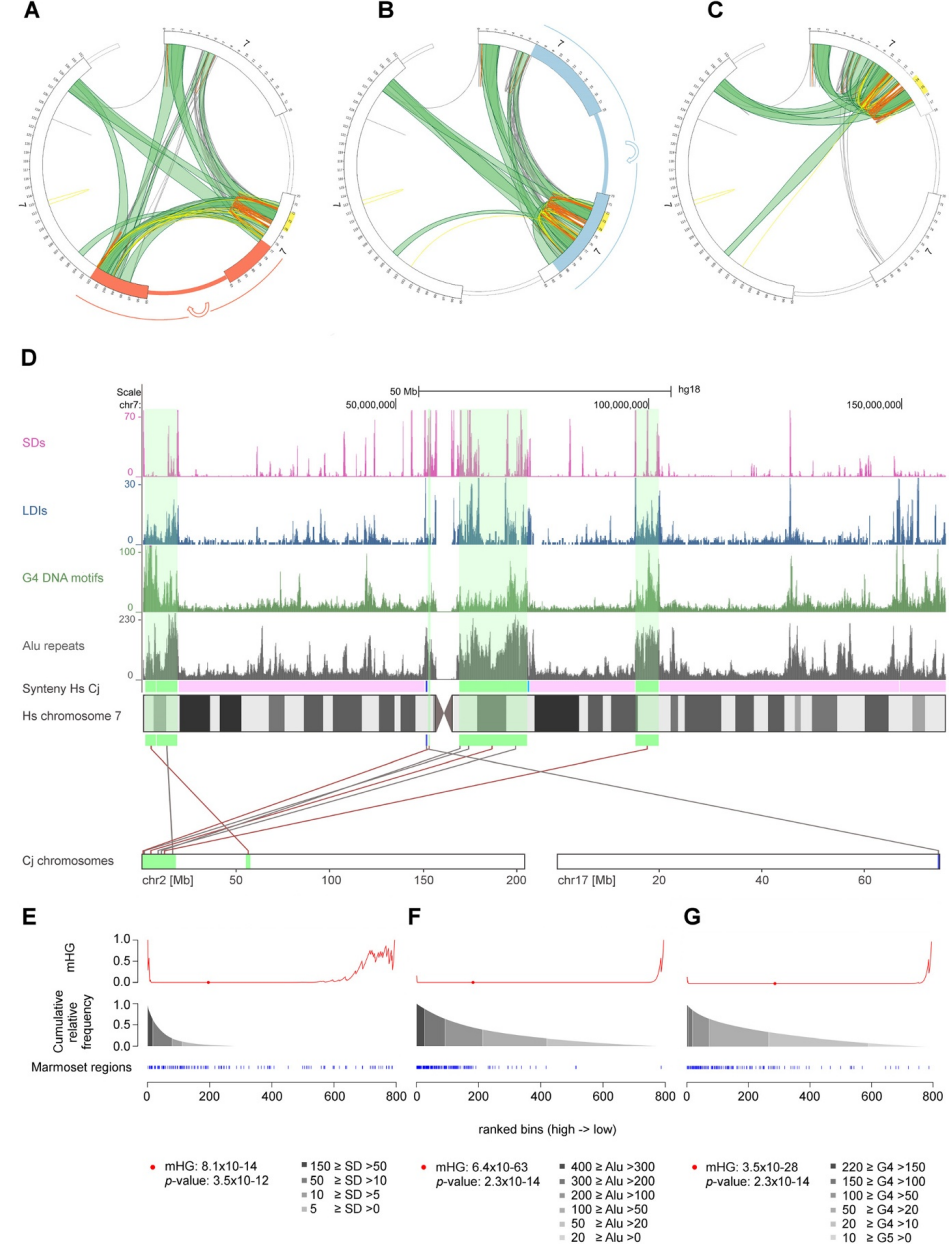
**Long distance interactions of human chromosome 7 connect sequences syntenic to the most proximal 17.9 Mb of marmoset chromosome 2 and cluster in regions rich in SDs, Alu repeats and G4 motifs. A-C)** Circos plots showing the patterns of long distance interactions (green bundles) in relation to SDs (following the colouring scheme of Figure 1) within the three segments of human chromosome 7 affected by the pericentric and paracentric inversions (as highlighted in blue in the idiogram of Figure 1); **(A)** before and **(B)** after *in silico* reversion of the paracentric inversion and **(C)** after reverting the pericentric inversion. The partial red and blue shading of the idiogram in **A** and **B** indicates the genomic interval inverted by the paracentric and pericentric inversion, respectively. **D)** distribution of SDs, long distance interactions (LDIs), G4 motifs and Alu repeats across human (Hs) chromosome 7 (100 kb bins) and its relation to marmoset (Cj) chromosome 2 syntenic regions (green blocks). Pink blocks highlight sequences syntenic to regions of marmoset chromosome 8. **E-G)** enrichment of SDs, Alu repeats and G4 motifs within chromosome 7 segments homologous to sequences of marmoset chromosome 2 (highlighted in blue). Chromosome 7 segments (binned in 200 kb windows) are displayed in ranked order according to feature count. The red curve and red dot above each plot indicate the hypergeometric score and its minimum (mHG), respectively.

SDs preferentially map to regions that are rich in long distance interactions. At the same time they are known to accumulate at synteny breakpoints [23, 25, 42]. This prompted us to search for particularities of long distance interaction patterns with respect to evolutionary breakpoints. We have focused on two recent rearrangements of chromosome 7 that have occurred during hominoid evolution and are not present in the homologous chromosome of orang-utan, a pericentric inversion in the common ancestor of human/gorilla followed by a paracentric inversion in the human/chimpanzee ancestor. As depicted in Figure 2A-C, synteny breakpoints coincide with changes in the characteristics of interaction patterns. To mimic the linear order of segments in gorilla and orang-utan we then recalculated the genomic coordinates of human chromosome 7 based on the fine-mapped evolutionary breakpoints (human/orang-utan, see Additional file 6). Figure 2A-C visualise the evolutionary split and relocation of a compact segment to three distant chromosomal regions in human and shows that these three - formerly adjacent - segments remain connected by long distance interactions. These segments comprise almost all sequences of human chromosome 7 that are syntenic to a large block (17.9 Mb) of marmoset chromosome 2 (Figure 2D; Ensembl v67 [43]). Genomic bins covering sequences of marmoset chromosome 2 were significantly overrepresented in regions rich in SDs as indicated by low probability scores based on minimum hypergeometric statistics [44] (*p*-value = 3.5 × 10^−12^; Figure 2E). Similarly a significant enrichment was detected in regions with a high frequency of Alu repeats (*p*-value = 2.3 × 10^−14^; Figure 2F), as well as G4 DNA motifs (*p*-value = 2.3 × 10^−14^; Figure 2G).

### Chromatin organisation of the Williams-Beuren region

**Figure 3:**
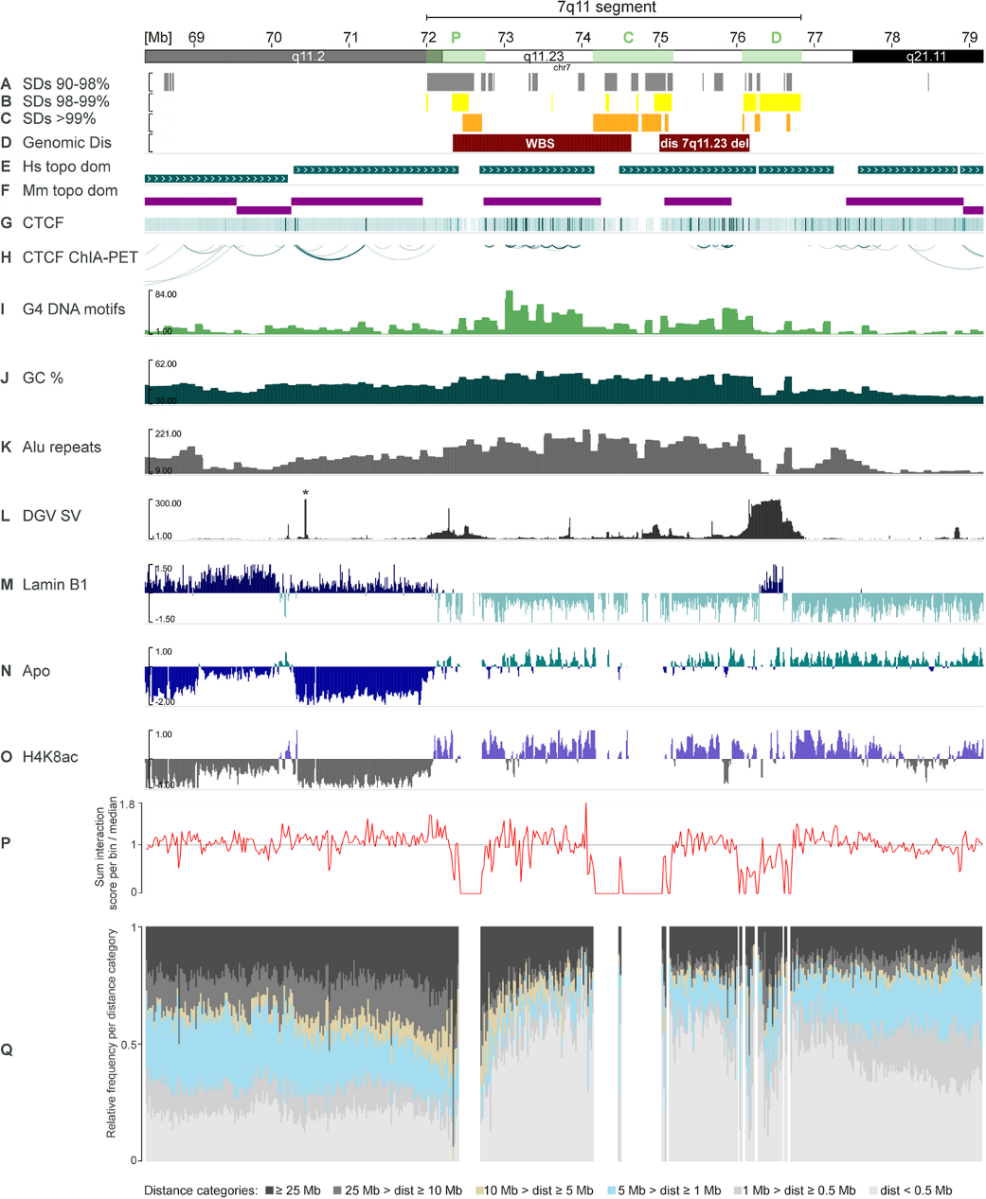
**Higher order chromatin organisation and SD localisation around the Williams-Beuren syndrome region.** All data are referring to genome release hg19 and are derived from IMR90 unless indicated otherwise. The proximal, central and distal SD clusters (P, C, D) of the 7q11 segment encompassing 4.8 Mb are highlighted in green within the chromosome banding track. **A-C)** localisation of SDs; colouring according to sequence similarity; grey: <98%, yellow: 98%-99%; orange: >99%; **D)** genomic interval commonly deleted in WBS and the distal 7q11.23 deletion syndrome; **E)** topological domains as defined by Dixon et al. [36]; **F)** topological domains identified in the corresponding region in mouse [36] after conversion to human hg19. Note that the murine topological domain homologous to sequences deleted in the distal 7q11.23 syndrome is not fully represented due to a break of synteny within this genomic interval. See Figure 4 for details; **G-H)** heatmap and arc view of CTCF binding sites as detected by ChIA-PET in MCF7; **I)** number of G4 motifs/100 kb bins; **J)** average GC-content within 100 kb bins; **K)** number of Alu repeats/100 kb bin; **L)** number of structural variants as annotated by Database of Genomic Variance (DGV) [104], *maximum of 1080 CNVs not shown; **M)** log2 ratio scores of the LaminB1 DamID Map (Tig3 cells) as reported by Guelen et al. [45]; **N)** log2 ratio scores of DNA regions prone to early apoptotic DNA degradation in 20 kb windows, turquoise: degraded DNA segments; **O)** log2 ratio scores of H4K8 acetylation profile in 20 kb windows, blue: hyperacetylation, grey: hypoacetylation; **P)** red curve representing the sum of all intrachromosomal interaction counts/bin divided by the median number of interactions for all bins of chromosome 7; **Q)** percentage of interactions categorised according to their interaction span size**;** light grey: <0.5 Mb, grey: 0.5-1 Mb, light blue: 1–5 Mb, light brown: 5–10 Mb, dark grey: 10–25 Mb, black: ≥25 Mb. Gaps in this plot are due to alignment problems of Hi-C data in regions harbouring SDs with high sequence similarity.

One of the three segments affected by the evolutionary rearrangement described above – the most closest segment to the centromere - contains three SD clusters (indicated by green boxes in the idiogram track in Figure 3), two of which are involved in the aetiology of the Williams-Beuren syndrome (WBS). Together these three SD clusters are encompassed by a 4.8 Mb genomic interval at 7q11.22-q11.23 (see Figure 3) (in the following named 7q11 segment). The most proximal SD cluster in the 7q11 segment starts at a transition of heterochromatin to euchromatin as demonstrated by our H4K8ac ChIP data and corroborated by numerous public data sets on posttranslational chromatin modifications (a selection of them is displayed in Figure 3 and Additional file 7). This heterochromatin to euchromatin switch is accompanied by changed probabilities of DNA attachment to the nuclear membrane [45] (Figure 3) and is also reflected by altered characteristics of replication timing and DNA degradation during early phases of apoptosis. In general, and in line with the literature, genome-wide analysis of apoptotic DNA degradation revealed significant correlation with both lamina attachment (ρ = −0.62, *p*-value < 2.2 × 10^−16^; Additional file 8) and replication timing (ρ = 0.65, *p*-value < 2.2 × 10^−16^) as defined by Spearman’s rank correlation test (Additional file 7). The patterns of apoptotic DNA degradation and its correlation to H4K8 acetylation were highly reproducible between two different cell lines (Additional file 9).

Given the reported association of gene density and chromatin organisation [46], we compared gene distribution and intron size inside and outside of the 7q11 segment. Gene density in the genomic region of this segment is higher than in 100000 randomly simulated intervals of chromosome 7 (23.86 vs. an average of 9.38 genes per Mb, estimated *p*-value < 0.0441). This difference in gene density was even more pronounced when focusing on the immediate genomic neighbourhood of the 7q11 segment; regions 4.8 Mb upstream and downstream contain an average of 1.45 genes per Mb (*p*-value = 5.829 × 10^−14^, two-tailed Fisher’s exact test) and 5.19 genes per Mb (*p*-value = 4.661 × 10^−7^, two-tailed Fisher’s exact test), respectively. At the same time, intron size of the 7q11 segment is decreased when compared to the average of 100000 simulations (3760 vs. 9827 bp, estimated *p*-value < 0.0453) and to the same number of genes (as located within the 7q11 segment) upstream and downstream of the segment (13772 and 9420 bp, *p*-value < 2.2 × 10^−16^, two-tailed Fisher’s exact test).

GC-content is another aspect that is tightly linked to chromatin conformation. GC-content within the 7q11 segment is 47.5% on average with a standard deviation of 4.4% based on 100 kb windows. We observed a considerable drop of GC-content (down to 36.3%) within the most distal SD block and public data suggest that this interval of about 295 kb is located next to the nuclear membrane if mapped correctly. G4 motifs show variable enrichment within the 7q11 segment, which is most prominent outside the SD blocks. We also observed a relative depletion of G4 motifs within the central block of SDs which is not reflected in a corresponding change of GC-content (Figure 3).

Next we have asked whether this distinct DNA conformation is also reflected in the Hi-C data set. The classification of Hi-C interaction data referring to chromosome 7 into six categories based on their interaction span size (ranging from less than 0.5 Mb to greater than 25 Mb) revealed that the change of chromatin state close to the WBS locus is also reflected by an increased proportion of interactions spanning less than 0.5 Mb (Figure 3), predominantly at the expense of interactions between 0.5-5 Mb and 10–25 Mb. This shift of span size characteristics is not accompanied by a general decrease of absolute interaction frequencies (red curve in Figure 3) and also lacks any symmetry around the gaps (owed to SDs with high sequence similarity) within the Hi-C data set, which would be expected if the observed changes in average span size are a consequence of mapping problems associated with the presence of SDs (Figure 3).

**Figure 4:**
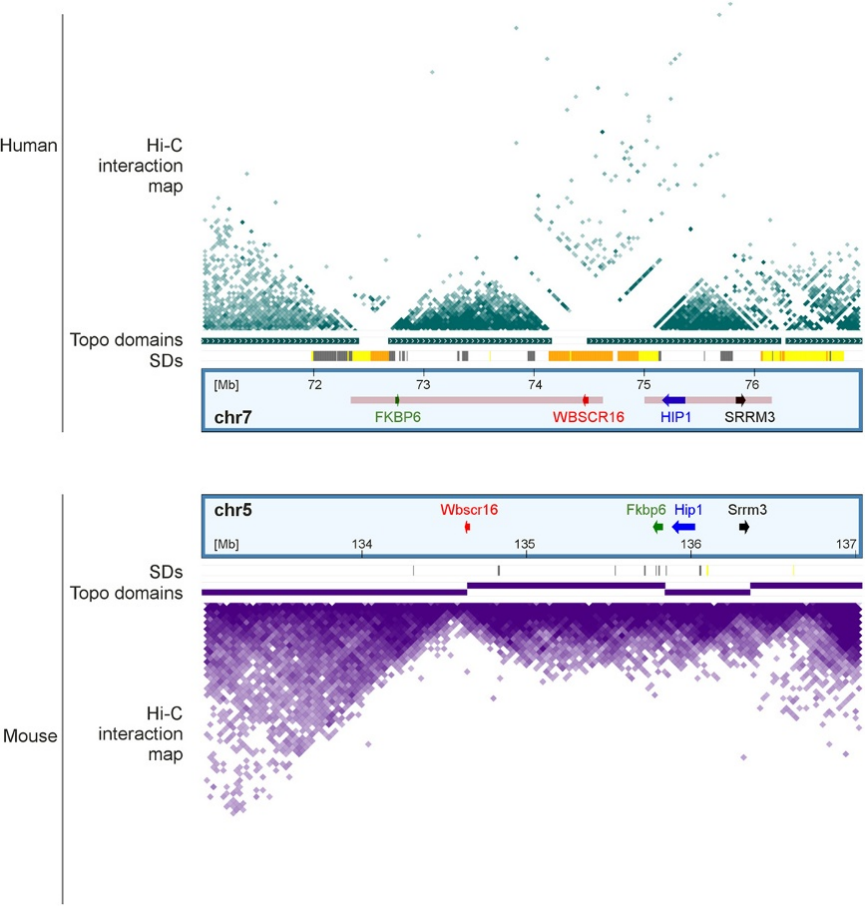
**Cross-species comparison showing that SDs next to the WBS locus have inserted at topological domain borders.** Hi-C interactions and topological domains in the human fetal fibroblast cell line IMR90 are shown in dark green in the upper part as triangle view and bars, respectively. SDs with sequence similarity of 98%-99% and above 99%, respectively, (shown in yellow and orange in the SDs track) coincide with gaps within the Hi-C data. SD distribution and Hi-C data of the corresponding region in mouse are given in the lower part of the image. The position of *FKBP6* and *WBSCR16*, the human orthologues of the two genes next to the murine topological domain borders are highlighted in green and red, respectively. The intervals commonly affected in WBS and the distal 7q11.23 syndrome are indicated by pale red bars. Note that the region distal to *SRRM3* including the distal SD block are homologous to a different mouse chromosome.

Furthermore, Hi-C interaction patterns suggest that the recurrent deletion involved in the aetiology of WBS removes one topological domain, which is flanked by SDs with highest sequence similarities. In order to validate this assumption and to rule out that domain border definition at this site simply reflects sequence read depletion in large SD blocks, we performed an interspecies comparison of the human WBS locus and its homologous region in mouse. Topological domains were reported to have a high degree of evolutionary conservation. Indeed, the corresponding region in mice (5qG2) comprises a distinct topological domain and the large SD blocks present in humans have inserted at sites that are homologous to murine topological domain borders (Figure 4).

## Discussion

In this study we have investigated the relation between chromatin organisation of human chromosome 7 and the distribution of segmental duplications.

Our study reveals that SDs preferentially map to those regions of chromosome 7, that are homologous to a 17.9 Mb large segment of marmoset chromosome 2. In the course of evolution, this formerly compact chromosomal segment split up and relocated to human chromosome 7p22, 7q11 and 7q22 by a pericentric and paracentric inversion in the common ancestor of human/gorilla and human/chimpanzee, respectively [47, 48]. Our analysis indicates that, despite these structural rearrangements, the three regions have retained their nuclear neighbourhood. This observation corroborates findings of evolutionarily conserved principles of nuclear organisation at the resolution of interphase FISH [49] and is in line with a recent report on an increased Hi-C interaction probability between murine syntenic breakpoint regions on human chromosomes, a phenomenon which has been termed spatial synteny [50]. As a consequence of spatial synteny, SD paralogs that are separated by structural rearrangements and appear distant on the linear chromosome are still in close spatial proximity in the interphase nucleus.

### A possible role for SDs in spatial synteny

In light of the observed conservation of nuclear architecture, we have asked what factors could account for spatial synteny and whether the biased distribution of SDs might play a role therein and in nuclear organisation in general [51, 52]. It is still unclear whether nuclear architecture is determined by a nuclear scaffold or represents the outcome of self-organisation choreographed by intrinsic properties of the chromatin itself (reviewed in [53]), or a combination thereof. Although several DNA-protein interactions and epigenetic marks clearly correlate with specific features of chromatin organisation, DNA sequence by itself is likely to play a crucial role [36, 53–55]. One DNA sequence feature significantly enriched in those segments of chromosome 7 that are syntenic to a large block of marmoset chromosome 2 are G4 DNA motifs (G_≥3_N_x_G_≥3_N_x_G_≥3_N_x_G_≥3_) [56] (Figure 2D and G). These motifs can establish highly stable intramolecular and intermolecular connections via Hoogsteen pairing between four guanines and have already been implicated in telomere organisation and in meiotic chromosome pairing [56–59]. The non-random distribution of G4 motifs along human chromosome 7, as shown in this study, could point at a possible function of quadruplex structures in the retention of spatial proximities also in interphase nuclei. High frequency of Alu repeats is another, partly interrelated, sequence feature that we have found significantly enriched in these highly interacting regions (Figure 2D and F). Alu repeat distribution is not the result of regional insertion preferences, but more likely the consequence of selective pressure on GC-content biased removal [60–63]. Against this background, Alu repeats have been implicated in higher order chromatin organisation [64, 65]. However, the overall presence of both Alu repeats and G4 motifs throughout the genome raises the question how such a sequence-directed organisation of the nucleus might obtain its specificity in the first place. The observed spatial proximity of SD paralogs (Figure 1), as well as their preferential insertion within Alu repeat and G4 motif-rich areas [18] (Figure 2) makes SDs ideal candidates to introduce sequence specificity into this process. For example, temporal somatic pairing could influence polymer dynamics and in this way accelerate the establishment of higher order chromatin organisation. Allelic or ectopic somatic pairing of homologous sequences is a widespread phenomenon in eukaryotes that is known to impact gene regulation and nuclear architecture ([66, 67], reviewed in [68]). Chromosomal structures enriched for interchromosomal SDs such as the telomeres and centromeres have already been reported to colocalise in interphase nuclei [69–74]. Notably, paralogous SDs show a remarkably high rate of interlocus gene conversion [75], which may indicate a high contact probability within the nucleus.

### SD distribution at the heterochromatin to euchromatin boundary at 7q11.22

Previous studies have reported the occurrence of SDs at the transition of heterochromatin to euchromatin [76–78]. This prompted us to re-evaluate the distribution of SDs in the context of new models of chromatin organisation, particularily the concept of topological domains. These megabase sized domains of highly interacting chromatin are remarkably stable between different cell types and highly conserved between mice and humans [36]. We have focused on the three SD blocks localised at the border of 7q11.22 to 7q11.23. These SDs are of special interest to human geneticists as non-allelic homologous recombination between them underlies the development of Williams-Beuren syndrome (WBS, OMIM 194050), the 7q11.23 duplication syndrome (OMIM 609757 [79]), the inversion that predisposes to the WBS deletion [80] and the distal 7q11.23 deletion syndrome (OMIM 613729 [81]).

Several observations indicate that the 7q11 segment containing these three SD blocks has a particular DNA conformation. This segment meets all criteria that have been defined for RIDGES (regions of increased gene expression; [82]), i.e. highly transcribed, GC-rich and gene-rich sequences with short introns and a high content of Alu repeats. RIDGES have a different degree of DNA compaction as suggested by computational analysis [83], an assumption, which is backed by the fact that the genomic characteristics of RIDGES largely overlap with those recently defined for DNA domains in an underwound state [84]. One factor for establishing and maintaining this specific chromatin conformation in this highly transcribed region may be G4 motifs, which are frequent in the 7q11 segment and have been reported to stabilise open chromatin [85]. Remarkably, sequences covered by the central and the distal SD cluster in the 7q11 segment show less G4 motif density and thus disrupt the continuity of G4 motif enrichment. Proceeding on the assumption that sequence reads were mapped unequivocally, the most distal SD block also has a high probability of being attached to the nuclear membrane (Figure 3).

Evaluation of CTCF interaction characteristics and the re-analysis of Hi-C data with focus on average interaction span sizes mirrors the particularities of chromatin conformation in the 7q11 segment (Figure 3). Moreover, Hi-C data [36] suggest that the genomic interval typically deleted in WBS patients comprises a distinct topological domain, which is flanked by SDs at its borders. Clearly, the paucity of Hi-C data mapping to SDs with highest sequence similarities complicates the interpretation of SD-related interaction patterns and may have compromised the precise definition of topological domains. In search of strategies which could enable us to discriminate SD-associated technical artefacts from biological relevant SD insertion at domain borders, we exploited the facts that topological domains are highly conserved between mice and humans [36] and that the syntenic region in mice lack these large SD blocks [23, 27, 86]. Our cross-species comparison revealed that the single copy sequences deleted in WBS indeed compose a distinct topological domain in mice, and that the large SD blocks present in humans have inserted at sites homologous to the murine domain borders. This insertion of DNA sequences with different characteristics, for example in terms of G4 motif density or preference for attachment to the nuclear membrane (see Figure 3), could emphasise the separation of topological domains. Thus SDs may impact chromatin organisation at the level of topological domains in a way which is reminiscent of what has been proposed for pericentric SDs at the chromosomal level, namely to facilitate differential gene regulation and to protect from the regulatory influence of adjacent sequences [19, 20]. The reciprocal event, a deletion of domain borders and linker region, has already been shown experimentally to provoke significant changes in the interaction pattern of two adjacent topological domains [87]. Further support for this assumption is provided by recent reports on the impact of WBS deletions on the interaction patterns of its adjacent topological domains [88].

Interestingly, although many SDs show accelerated rates of sequence divergence [26], SDs involved in the aetiology of WBS and several other genomic disorders show a considerably high rate of gene conversion, which preserves their sequence similarity [89–92] and, as a consequence, the risk of recombination events that cause the genomic disorder [93, 94]. On one hand, recurrent recombinations of paralogous SDs, which cause the high rate of intrachromosomal deletions and inversions in the WBS region, supports the assumption of a high contact probability between these paralogous SDs within the nucleus. On the other hand, it raises the question whether sequence similarity might serve a function that could compensate for the associated high susceptibility to structural rearrangements mediated by SDs with high sequence similarity. For example, SDs could influence chromatin organisation by somatic pairing as discussed above or by RNA-based mechanisms. The latter option would be one explanation for the reported high transcriptional activity of pseudogenes mapping to SDs [4], with many of them regulated in a tissue-specific manner [17]. Notably, the frequent interaction of the Prader-Willi syndrome imprinting centre (15q13) with two adjacent SDs has already inspired discussions on the functional impact of SDs on chromatin organisation [95].

## Conclusions

Our study suggests a link of nuclear architecture and the propagation of SDs across chromosome 7. Higher contact probabilities could promote regional SD insertion, but also could be a factor of nuclear organisation themselves, which promotes their propagation and evolutionary fixation in the genome.

## Methods

### Analysis of long distance interactions

We have downloaded normalised intrachromosomal Hi-C data (hg18) of autosomes with 20 kb resolution derived from the human fetal lung fibroblast cell line IMR90 (replicate 1; [36]). A stringent cut-off was used to remove interaction (IA) bins represented by less than 15 independent sequence counts. Long distance interactions of chromosome 7 were defined by a minimal span size of 25 Mb. “Circos utilities/bundlelinks” [40] was employed to fuse long distance interactions to one bundle when at least five interaction bins were within a maximum distance of 500 kb at the start and target sites. We applied different combinations of filter options in terms of interaction counts per bin (at least 10, at least 15, and 10–50 IA/bin) and minimum span sizes (10 and 25 Mb) to evaluate the impact of thresholds on the bundle pattern (see Additional files 1 and 4). Moreover, we introduced a third filter based on the overlap of a given bin with SDs in order to correct for interactions that are owed to erroneous sequence alignments. BEDTools ”pairToPair” [96] was used to remove all interaction bins that connect two SD paralogs (removed IA bins: n = 159) or that overlap with any SD at all (removed IA bins: n = 126883) (see scheme in Additional file 4I). The remaining interactions were bundled using adapted criteria to factor the reduced number of interactions in total.

Beside this filtering of Hi-C data on the level of genomic bins covering SDs we have repeated our filtering and bundling analysis on the level of paired-end reads mapping to SD regions. On the basis of the method of SUNs (Single Unique Nucleotides) discovery [97] we merged all regions covered by SDs, divided them into 30 bp long reads and remapped them to the human reference genome using RazerS 3 [98]. 30mer alignments mapping only once and with a maximum edit distance of 2 bp were considered as unique sequences. This data set was used to filter out ambiguously mapped paired-end reads within the Dixon data set mapping to these regions. The remaining read pairs were binned into 20 kb genomic windows and the resulting observed interaction counts per bin were re-normalised using the expected contact probability for the unfiltered read pairs as calculated by hicpipe [41]. The re-normalised interaction bins were filtered for long distance interactions (at least 15 interaction counts per bin, spanning more than 25 Mb) and these were bundled applying the criteria described above. Long distance interaction bundles were visualised by means of Circos plots [40].

### Public data sets

Our analysis took advantage of various publicly available data sets (segmental duplications [5, 86], [36, 45, 99–105], GSM935404, GSM970215, GSM469974, GSM469968, GSM521915, GSM521900, GSM469970, GSM521884, GSM521883, GSM521897, GSM469966, GSM521890, see Additional files 10 and 11 for details), which were downloaded from the UCSC Table Browser [106], the annotation database of the UCSC Genome Browser [107], the non-B database [100] and from the website given in Dixon et al. [36].

### SD distribution and intrachromosomal interaction patterns

Segmental duplications of all sequence similarities have been categorised in those with their paralog mapping exclusively to the same chromosome (intra) and in those with their paralog mapping intrachromosomal and genome-wide. Additionally, in line with the colouring scheme used in the UCSC Genome Browser [108] segmental duplications have been categorised in those with sequence similarities below 98% (grey), between 98% and 99% (yellow) and above 99% (orange), respectively, and all three categories combined. Enrichment of the above-mentioned SD categories within long distance interaction bundles was tested. For this purpose the base pair overlap of SD covering regions of chromosome 7 with the bundle intervals of chromosome 7 (data set obtained with the cut-offs: >15 interaction counts/bin, interaction distance > 25 Mb) was determined and compared to 10000 random intervals employing the following strategy. First, to combine overlapping intervals within a given SD or bundle data set, respectively, the BEDTools “mergeBed” [96] was used. Second, the base pair overlap of SD data sets with long distance interaction bundles was calculated (observed base pair overlap) (BEDTools "coverageBed"). As control a resampling of the SD categories was performed (10000×; BEDTools "shuffleBed") with the following conditions for the random intervals: locate to the same chromosome and with the same interval sizes as the input SD data set, non-overlapping intervals and exclusion of annotation gaps. Subsequently the base pair overlap for each of the 10000 random data sets with the long distance interaction bundles was calculated (expected base pair overlaps). The fold change of the observed base pair overlap was calculated as the ratio of observed base pair overlap and the mean of 10000 expected base pair overlaps. The number of expected base pair overlaps greater or equal to the observed base pair overlap was counted for each SD category and used to calculate the *p*-value as described for Monte Carlo resampling in [109]. The *p*-value adjustment was performed according to the Benjamini-Hochberg method. Histograms of the expected base pair overlaps for each SD category were drawn using the R package ‘ggplot2’ [110].

In addition, SD enrichment within interaction bundles (data set obtained with the cut-offs: >15 interaction counts/bin, interaction distance > 25 Mb) was determined for all chromosomes using SDs with paralogs exclusively mapping to the same chromosome, or intrachromosomal and genome-wide.

Finally, SD enrichment within regions where bins are part of all bundle data sets (obtained by intersection of all twelve data sets resulting from different filter criteria, see Additional file 3) was calculated using SDs with paralogs mapping intrachromosomal and genome-wide.

### Fine-mapping of evolutionary breakpoints and mimicking interaction patterns in orang-utan and gorilla

Alignments were retrieved from the Ensembl database (version 67) using the Perl API [43]. As the paracentric inversion is not represented in the current version of the gorilla genome (*Gorilla gorilla gorilla;* gorGor3.1; May 2011), the proximal and distal breakpoint of both inversions were determined by plotting the orang-utan genome (*Pongo abelii;* WUGSC2.0.2/ponAbe2; July 2007) versus the human genome (GRCh37/hg19; February 2009). A corresponding dot plot, which uses the UCSC colouring scheme for the chromosome numbers is shown in Additional file 6. Segmental duplications were superimposed onto the dot plot following the colouring scheme introduced above (Additional file 6). The fine-mapped coordinates of the paracentric and pericentric inversion of chromosome 7 derived from this analysis (para: chr7:76646908 and chr7:102118853, peri: chr7: 6875820 and 80857936; hg18) were used to recalculate the genomic coordinates of long distance interactions and SDs in order to mimic the situation in gorilla and orang-utan. The three segments surrounding the evolutionary breakpoints, the positional changes of SDs and long distance interactions after *in silico* reversion were visualised by means of Circos plots [40].

### Synteny of human chromosome 7 and enrichment analysis for SDs, Alu repeats and G4 motifs

Syntenic regions of human chromosome 7 and marmoset (*Callithrix jacchus*) were obtained from Ensembl database (version 67) [43] and converted to hg18 coordinates using the default settings of the LiftOver tool [108]. We divided chromosome 7 into 200 kb bins (n = 795), of which 125 comprise sequences homologous to marmoset chromosome 2. The minimum hypergeometric score and its exact *p*-value were calculated as described by Eden et al. [44]. In brief, we have shuffled the natural order of genomic bins in order to minimise the influence of the genomic order of bins with identical values. Then we ranked all bins in ascending order according to their counts for the respective feature (Alu, SD, G4). The enrichment of marmoset chromosome 2 sequences within the highest scoring bins was quantified by means of the hypergeometric score and the *p*-value was calculated for the minimum hypergeometric score (mHG). Distribution of SDs, long distance interactions, G4 DNA motifs, Alu repeats and syntenic regions of human chromosome 7 and marmoset were visualised in the UCSC Genome Browser [108] (upper part in Figure 2D) and combined with further information on synteny derived from the Ensembl Genome Browser (lower part in Figure 2D).

### Chromatin immunoprecipitation

Human fetal lung fibroblast cell lines IMR91L (male) and IMR90 (female) were obtained from the Coriell Institute for Medical Research. Both cell lines were cultured in Eagle´s minimum essential medium (EMEM) supplemented with 10% fetal bovine serum (Sigma-Aldrich, Saint Louis, USA), 2 mM UltraGlutamine 1 (Lonza, Walkerville, USA), 1 mM sodium pyruvate and 100 units/mL penicillin/streptomycin. The fibroblasts were maintained at 37°C with a humidified atmosphere of 5% CO_2_ and ambient oxygen. Chromatin immunoprecipitation was done according to the Transcription Factor ChIP kit protocol (Diagenode, Liège, Belgium). In brief, lysed cells were sonicated using the Bioruptor UCD-200 device (Diagenode, Liège, Belgium), followed by overnight incubation of 1 × 10^6^ cells with 5 μg of antibody against Histone H4 lysine 8 acetylation (pAb-103-050; Diagenode, Liège, Belgium). The subsequent chromatin reverse crosslinking, elution and purification of ChIP DNA and input DNA were done employing the IPure Kit (Diagenode, Liège, Belgium).

### Analysis of DNA degradation during early phases of apoptosis

Apoptosis of IMR90 and IMR91L cells was induced by exposing 2 × 10^6^ cells to either 1 μmol/L staurosporine (Cell Signaling Technology, Inc., Danvers, USA)/0.1% DMSO or 0.1% DMSO alone (as control) for four hours at 37°C. An aliquot of about 5-10 × 10^6^ cells/mL was co-stained with Annexin V-APC (BD Biosciences, San Jose, USA) and 7-Aminoactinomycin D (7-AAD, BD Biosciences, San Jose, USA) for 15 minutes to monitor the progress of apoptosis by FACS analysis.

The remaining cells were treated with lysis buffer (0.40 M Tris–HCl pH 8.0, 0.06 M Na-EDTA, 0.15 M NaCl, 1% SDS) and RNA was digested for 1 hour at 37°C using 15 μg/mL RNase A. 1 M sodium perchlorate and one volume chloroform were added to deproteinise cell lysates. DNA fragmentation was checked using the Genomic DNA Screentape on an Agilent 2200 Tap2station (Agilent, Santa Clara, USA) (see Additional file 9).

High molecular (>48 kb) and degraded apoptotic DNA (~4 kb) were extracted by cutting slices out of a preparative 1% low melt agarose gel and subsequent digestion with β-Agarase I according to the manufacturer´s protocol (New England Biolabs, Ipswich, USA).

### Microarray hybridisation

Purifed DNA from ChIP and apoptotic DNA degradation experiments were amplified by means of the GenomePlex Whole Genome Amplification Kit (Sigma, Saint Louis, USA). Regional preferences in apoptotic DNA degradation and H4K8 acetylation were determined by co-hybridising high molecular (>48 kb) and degraded apoptotic DNA (~4 kb), and ChIP DNA and input DNA onto a 400 k whole genome oligonucleotide array (GPL9777) and region-specific custom oligonucleotide array covering the interval chr7:69936560–70795513 (hg19) with an average oligospacing of 198 bp (GPL17964), respectively (following the protocols for array CGH provided by the manufacturer (Agilent, Santa Clara, USA)). Image analysis, normalisation and annotation were done with Feature Extraction 10.5.1.1 (Agilent, Santa Clara, USA) using the default settings. Data visualisation and further analysis was performed with GenomeCAT (Tebel et al., manuscript in preparation; http://www.molgen.mpg.de/204904/GenomeCAT) and the Human Epigenome Browser [111, 112].

### RNA expression profiling

Expression profiling was performed by Next-generation sequencing on a SOLiD 5500xl Genetic Analyzer (Life Technologies, Carlsbad, USA). Total RNA was extracted from IMR91L cell cultures using TRIzol (Life Technologies, Carlsbad, USA). 10 μg of each total RNA sample was spiked with ERCC spike-in control mixes (Life Technologies, Carlsbad, USA) prior to removal of the rRNA by use of the RiboMinus Kit (Life Technologies, Carlsbad, USA). The RNA was then prepared for sequencing using the protocol and components provided with. In brief, the rRNA-depleted RNA was fragmented by chemical hydrolysis, phosphorylated and purified. Adaptors were then ligated and hybridised to the RNA fragments and reverse transcribed into cDNA. The cDNA was then purified and size-selected using two rounds of Agencourt AMPure XP bead purification (Beckman Coulters Genomics, Danvers, USA) and released from the beads. The sample was then amplified by 12 PCR cycles in a T3 Thermocycler (Biometra, Göttingen, Germany) in the presence of primers that contained unique sequences (barcoding) in order to determine the origin of the sequence after pooling of the fragments and sequencing. The size distribution and concentration of the fragments were determined with an Agilent 2100 Bioanalyzer (Agilent Technologies, Santa Clara, USA) and quantitative PCR using a LightCycler 480 Real-Time PCR System (Roche Applied Science, Penzberg, Germany) and the KAPA Library Quant ABI SOLiD kit (Peqlab Biotechnologie GmbH, Erlangen, Germany).

The cDNA fragments were then pooled in equimolar amounts and diluted to 61 pg/μL corresponding to a concentration of 500 pM. 50 μL of this dilution was mixed with a freshly prepared oil emulsion, P1 and P2 reagents and P1 beads in a SOLiD EZ Bead Emulsifier prepared according to the E80 scale protocol (Life Technologies, Carlsbad, USA). The emulsion PCR was carried out in a SOLiD EZ Bead Amplifier (Life Technologies, Carlsbad, USA) using the E80sm setting. To enrich for the beads that carried amplified template DNA, the beads were purified on a SOLiD EZ Bead Enricher using the recommended chemistry and software (Life Technologies, Carlsbad, USA).

The purified beads were then loaded onto a SOLiD 6-lane Flowchip and incubated upside down for 1 hour at 37°C. The Flowchip was then positioned in the 5500xl SOLiD System and the DNA was sequenced using 50 nucleotides in the forward direction and 35 nucleotides in the reverse direction and the recommended chemistry (Life Technologies, Carlsbad, USA).

Sequence reads mapping to RefSeq coding exons and matching the coding strand were counted towards coding RNAs, all other mapping reads were counted towards non-coding RNAs.

### Genomic characterisation of the Williams-Beuren region

Own experimental results and public data (Additional files 10 and 11) were conflated in the Human Epigenome Browser hosted by Washington University [111, 112]. Regional characteristics of lamin B1 interaction sites [45], replication timing [101, 102] and apoptotic DNA degradation (log2 ratio) were compared for 20 kb bins using Spearman's rank correlation test implemented in R [113].

For calculation of gene density and intron size of genes on chromosome 7 within the 7q11 segment or the intermediate neighbourhood, genomic coordinates of known canonical genes and their introns were downloaded from the UCSC Table Browser. Number of genes and intron length within each region were determined by means of “BEDTools/intersectBed” [96]. Gene density for each region was calculated as the number of genes per megabase. Statistical significance was estimated using 100000 random simulations or a Fisher’s exact test.

### Calculation of average span sizes of intrachromosomal interactions of chromosome 7

All intrachromosomal interaction bins of chromosome 7 indicated by at least one normalised interaction count between two genomic bins according to Dixon et al. [36] were categorised into six classes based on their span size: i) <500 kb, ii) 500 kb to less than 1 Mb, iii) 1 Mb to less than 5 Mb, iv) 5 Mb to less than 10 Mb, v) 10 Mb to less than 25 Mb and vi) span sizes equal or greater than 25 Mb.

For each bin and span size category we summed up the scores separately. The relative contribution of each category to the total score of interaction counts/bin was calculated by dividing the category score through the total score of each bin. For the purpose of comparability within Figure 3, genomic coordinates have been converted to hg19 using the default settings of the LiftOver tool [108].

### Topological domains in mice

Coordinates of mouse (mm9) topological domains were obtained from [36] and converted to hg19 using the default settings of the LiftOver tool [108]. Both the original and the converted mouse domains were visualised within the Human Epigenome Browser [112] in the mm9 and hg19 assembly, respectively. Orthologous genes located at the murine domain borders were plotted at the corresponding location in the human genome employing the Multi-Genome Synteny Viewer (mGSV) [114].

## Availability of supporting data

Microarray data generated in this study have been submitted to NCBI GEO (http://www.ncbi.nlm.nih.gov/geo/) under accession number GSE41356.

RNA sequencing data have been submitted to Sequence Read Archive (SRA) (http://www.ncbi.nlm.nih.gov/Traces/sra/) under accession number SRS366467.

## Electronic supplementary material

Additional file 1: **(A) Number of interaction bins before and after removing bins covering SDs and (B) the number of interaction bins and resulting bundles for all cut-offs for chromosome 7.** (XLSX 12 KB)

Additional file 2: **Distribution of segmental duplications (SDs) and non-bundled long distance interactions (>25 Mb) in relation to acetylation of H4K8, transcriptional activity and lamina associated domains on human chromosome 7 (derived from IMR90 unless indicated otherwise; layout is identical to Figure** 1 **, apart from the replacement of bundled interactions by the interaction bins before bundling).** A) H4K8 acetylation profile, dark yellow: hyperacetylation of H4K8; blue: hypoacetylation of H4K8. B) the red and blue curve represent RNA-seq read counts/100 kb bin for coding and non-coding RNA, respectively (IMR91L). C) grey areas underlying the two histograms mark lamina associated domains (LADs, Tig3 cells). D) idiogram of chromosome 7, the Williams-Beuren syndrome region is highlighted in yellow beside the idiogram (at 72–74 Mb, hg18). E) transparent blue shading of the idiogram illustrates the inversion-affected segments of chromosome 7 depicted in Figure 2A-C. Long distance interactions at 20 kb resolution (F) and segmental duplications (G) are depicted in the inner circle; green links: long distance interactions between genomic 20 kb bins; grey: SDs with sequence similarity <98%; yellow: SDs with sequence similarity 98-99%; orange: SDs with sequence similarity >99%. (PDF 3 MB)

Additional file 3: **SDs have a higher probability to be located within long distance interaction bundles.** The observed base pair overlap of bundle intervals of chromosome 7 (chr7), genome-wide (all chr) and the intersected bundle regions of all 12 cut-off data sets of chromosome 7 (intersected bundles) with SDs was compared to expected values obtained from 10000 random data sets. The eight plots depict the distribution of the base pair overlaps (in bp) of the bundle data sets with the resampled SD categories: for all SDs with paralogs mapping intrachromosomal and genome-wide (all SDs), all SDs with paralogs mapping exclusively intrachromosomal (all intra SDs) or for the three sequence similarity categories (SDs < 98%, SDs 98%-99%, SDs > 99% sequence similarity) separately. The observed value is indicated by a vertical dashed line (in red), with the enrichment score and its significance after correction for multiple testing given beside. (PDF 340 KB)

Additional file 4: **Changes of long range interaction patterns of human chromosome 7 as a consequence of filtering for interaction counts, span size and SD overlap.** Normalised Hi-C interaction data were filtered based on the number of interaction counts (IA) per bin, interaction distance and overlap with SDs. From a total of 12 data sets, only the results for the cut-off “>15 IA/bin” in combination with interaction distance and SD overlap are shown. The first two rows show the effect of using different interaction distances (>25 Mb and >10 Mb) on the interaction bundle pattern (green ribbons). The three columns compare the bundle patterns before and after removing bins covering SDs. The two strategies to exclude bins covering regions with SDs are displayed in I; (w/o - without). all IA bins (A-B): no exclusion of interaction bins based on overlap with SDs; IA bins w/o SD paralogs (C-D): interacting bins were excluded if they connect two paralogous SDs; IA bins w/o any SD (E-F): interaction bins were excluded if they overlap with any SD at all. Visualisation as described in Figure 1. The plot obtained with the filter options used for Figure 1 and Additional file 2 is marked by a grey triangle (A). G) depicts a hypothetical bundle pattern (blue ribbons), which is obtained by connecting all segments of human chromosome 7 that are homologous to marmoset chromosome 2 (highlighted in blue within the idiogram). H) the outer histogram displays the frequency that a given 20 kb bin is part of a bundle region after filtering with the 12 different combinations of cut-offs (red shading within histogram – regions where bins are part of all bundle data sets). Red bundles represent the triangular interaction pattern resulting from bundle regions that are shared by all 12 data sets obtained by various thresholds (see Methods) and additionally map to the same target sites. I) scheme for the removal of bins covered by SDs. J) pattern of long distance interactions after SD filtering at the resolution of paired-end reads. After exclusion of all non-unique paired-end reads, data were re-normalised. Bundling of the resulting interacting bins reproduced the triangular interaction patterns seen above. (PDF 2 MB)

Additional file 5: **All 20 kb bins of chromosome 7 and the respective relative number of data sets with a bundle region encompassing a given bin.** For example a value of “1” means that the bin is part of a bundle region in all twelve data sets, green shading highlights bins that are part of bundles depicted in Figure 1. (XLSX 217 KB)

Additional file 6: **Dot plots indicating the breakpoints of the paracentric and pericentric inversion.** A) Dot plot of human chromosome 7 (hg19) against the orang-utan genome (ponAbe2). B) zoom-in of the proximal and distal breakpoint (vertical red lines) of the paracentric inversion. See text for details. (PDF 650 KB)

Additional file 7: **Additional data on higher order chromatin organisation and SD localisation around the Williams- Beuren syndrome region as depicted in Figure** 3 **.** All data are referring to genome release hg19 and are derived from IMR90. A) chromatin states as defined by [115, 116]; light blue: heterochromatin, dark green: active transcription, yellow: enhancers, grey: weak repression by Polycomb, dark grey: strong repression by Polycomb, red: bivalent poised TSS; B) replication timing phases from G1 (top) to G2 (bottom; [101, 102] in black); C) markers for repressed chromatin (H3K9me3, H3K27me3, H4K20me1) in red; D) markers for active chromatin (H3K4me2, H3K4me3, H3K14ac, H3K18ac, H3K4me1, H3K27ac, H3K36me3) in green; E) CTCF binding sites; F) red bars indicating the intervals typically deleted in WBS and the distal 7q11.23 deletion syndrome; G) distribution of SD blocks following the colouring scheme as described in the Methods section; H) arc view highlighting corresponding SD paralogs with more than 99% sequence similarity; I) arc view highlighting corresponding paralogs for all SDs; J) topological domains defined by Dixon et al. [36]; K) two-dimensional heatmap based on Hi-C data from Dixon et al. [36]. (PDF 2 MB)

Additional file 8: **Comparison of lamina associated domain borders, acetylation of H4K8 and apoptotic DNA degradation.** Clustered heatmaps display the degree of H4K8 acetylation and apoptotic DNA degradation 500 kb upstream and downstream of the borders of lamina associated domains (LADs). Data are shown as 20 kb bins. Only LADs with a minimum size of 500 kb and a minimum distance of 500 kb to neighbouring LADs have been selected. DNA degradation and H4K8 acetylation profiles are shown with orange representing genomic regions with a higher rate of apoptotic DNA fragmentation and H4K8 acetylation, while blue depicts less degraded DNA segments and hypoacetylated H4K8, respectively. Visualisation by means of R packages ‘cluster’ [117] and ‘gplots’ [118]. (TIFF 170 KB)

Additional file 9: **Reproducibility of apoptotic DNA degradation patterns.** A) fragmentation of genomic DNA isolated from IMR90 cells treated with DMSO (control, in green) and after apoptosis induction using staurosporine (in pink) as visualised by an Agilent 2200 Tap2station (Agilent, Santa Clara, USA). B) for visualising the correlation between the IMR90 and IMR91L apoptosis data sets we performed a two-dimensional kernel density estimation ('MASS' R package [113, 119, 120]) of 100 kb binned data under omitting missing values and passed the output to the filled.contour function of the R package 'graphics'. Additionally H4K8 acetylation patterns of the same cell lines were compared; *p*-value for all plots < 2.2 × 10^−16^. (PDF 822 KB)

Additional file 10: **Publicly available data sets used in the study.** (DOCX 32 KB)

Additional file 11: **Data on posttranslational histone modifications used in the study.** (XLSX 10 KB)

## Abbreviations

IA: Interaction
NAHR: Non-allelic homologous recombination
SD: Segmental duplication
WBS: Williams-Beuren syndrome.
