# Supplementary figures and images for: Distribution of segmental duplications in the context of higher order chromatin organisation of human chromosome 7
Source: BMC Genomics. 2014 Jun 29;15:537. doi: 10.1186/1471-2164-15-537 (PMC4092221; doi:10.1186/1471-2164-15-537)

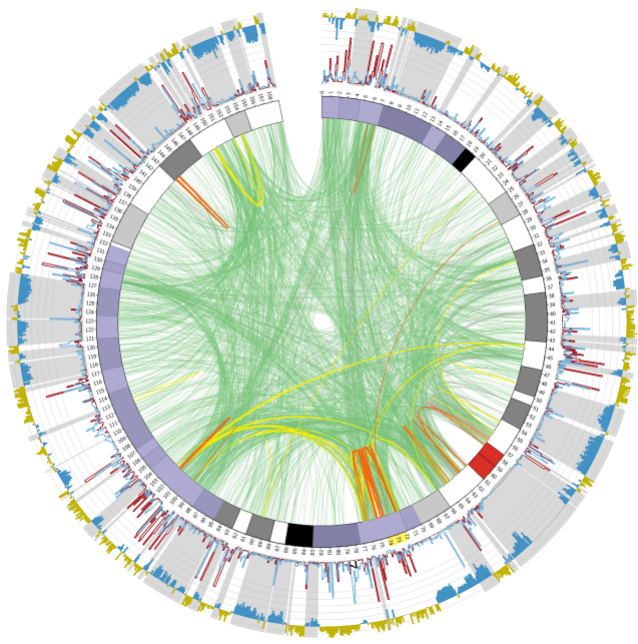

Supplement: Supplementary file 2 — Additional file 2: Distribution of segmental duplications (SDs) and non-bundled long distance interactions (>25 Mb) in relation to acetylation of H4K8, transcriptional activity and lamina associated domains on human chromosome 7 (derived from IMR90 unless indicated otherwise; layout is identical to Figure 1 , apart from the replacement of bundled interactions by the interaction bins before bundling). A) H4K8 acetylation profile, dark yellow: hyperacetylation of H4K8; blue: hypoacetylation of H4K8. B) the red and blue curve represent RNA-seq read counts/100 kb bin for coding and non-coding RNA, respectively (IMR91L). C) grey areas underlying the two histograms mark lamina associated domains (LADs, Tig3 cells). D) idiogram of chromosome 7, the Williams-Beuren syndrome region is highlighted in yellow beside the idiogram (at 72–74 Mb, hg18). E) transparent blue shading of the idiogram illustrates the inversion-affected segments of chromosome 7 depicted in Figure 2A-C. Long distance interactions at 20 kb resolution (F) and segmental duplications (G) are depicted in the inner circle; green links: long distance interactions between genomic 20 kb bins; grey: SDs with sequence similarity <98%; yellow: SDs with sequence similarity 98-99%; orange: SDs with sequence similarity >99%. (PDF 3 MB) [file 12864_2013_6219_MOESM2_ESM.pdf]

chr7

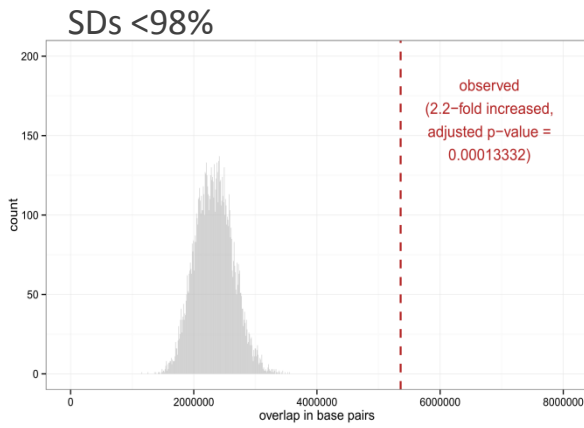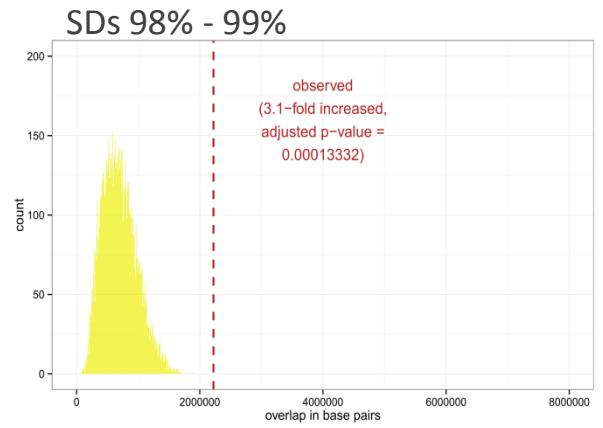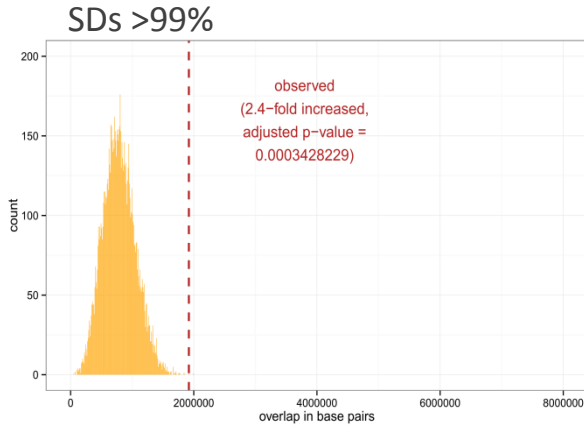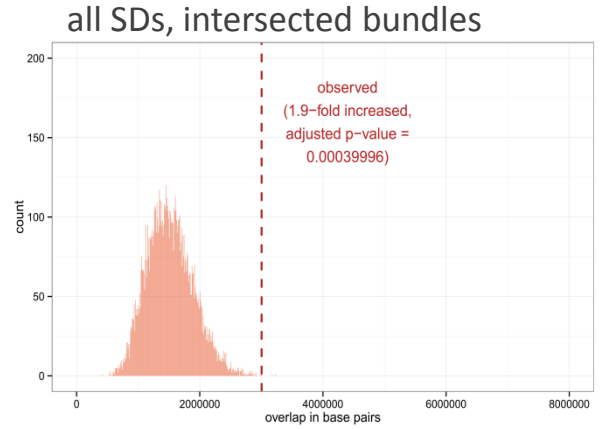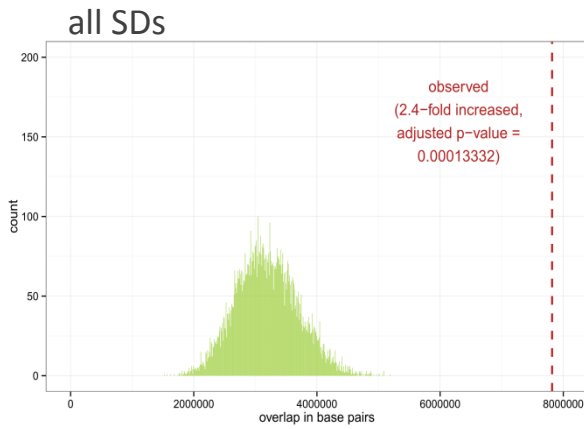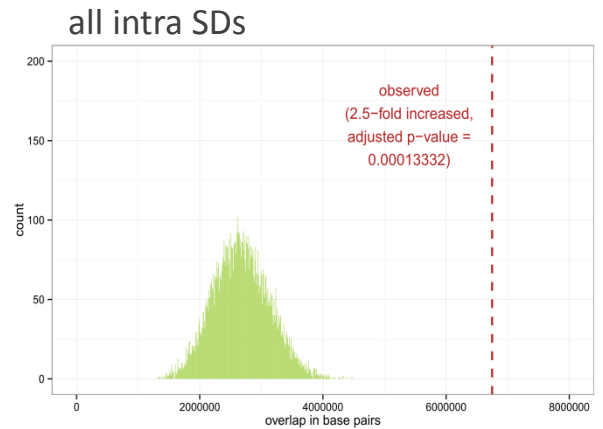

all chr

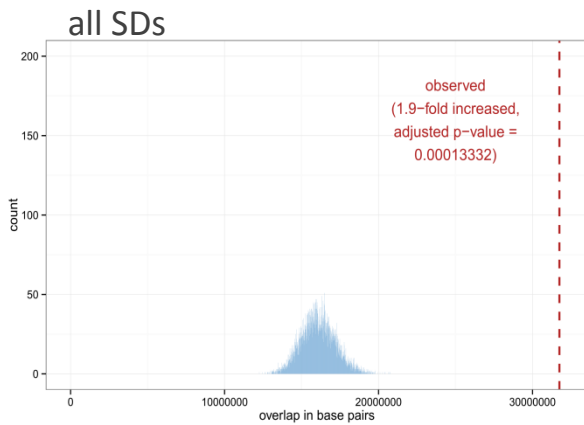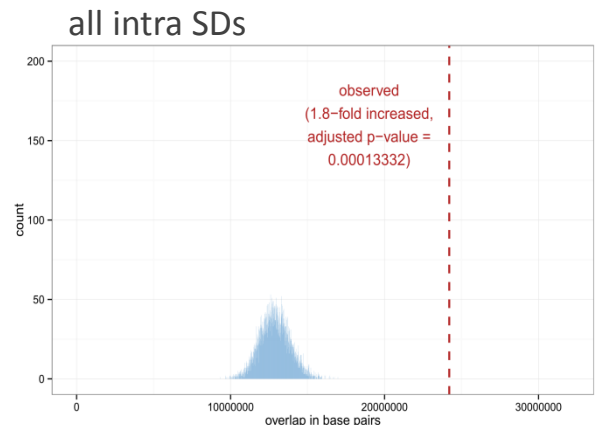

Supplement: Supplementary file 3 — Additional file 3: SDs have a higher probability to be located within long distance interaction bundles. The observed base pair overlap of bundle intervals of chromosome 7 (chr7), genome-wide (all chr) and the intersected bundle regions of all 12 cut-off data sets of chromosome 7 (intersected bundles) with SDs was compared to expected values obtained from 10000 random data sets. The eight plots depict the distribution of the base pair overlaps (in bp) of the bundle data sets with the resampled SD categories: for all SDs with paralogs mapping intrachromosomal and genome-wide (all SDs), all SDs with paralogs mapping exclusively intrachromosomal (all intra SDs) or for the three sequence similarity categories (SDs < 98%, SDs 98%-99%, SDs > 99% sequence similarity) separately. The observed value is indicated by a vertical dashed line (in red), with the enrichment score and its significance after correction for multiple testing given beside. (PDF 340 KB) [file 12864_2013_6219_MOESM3_ESM.pdf]

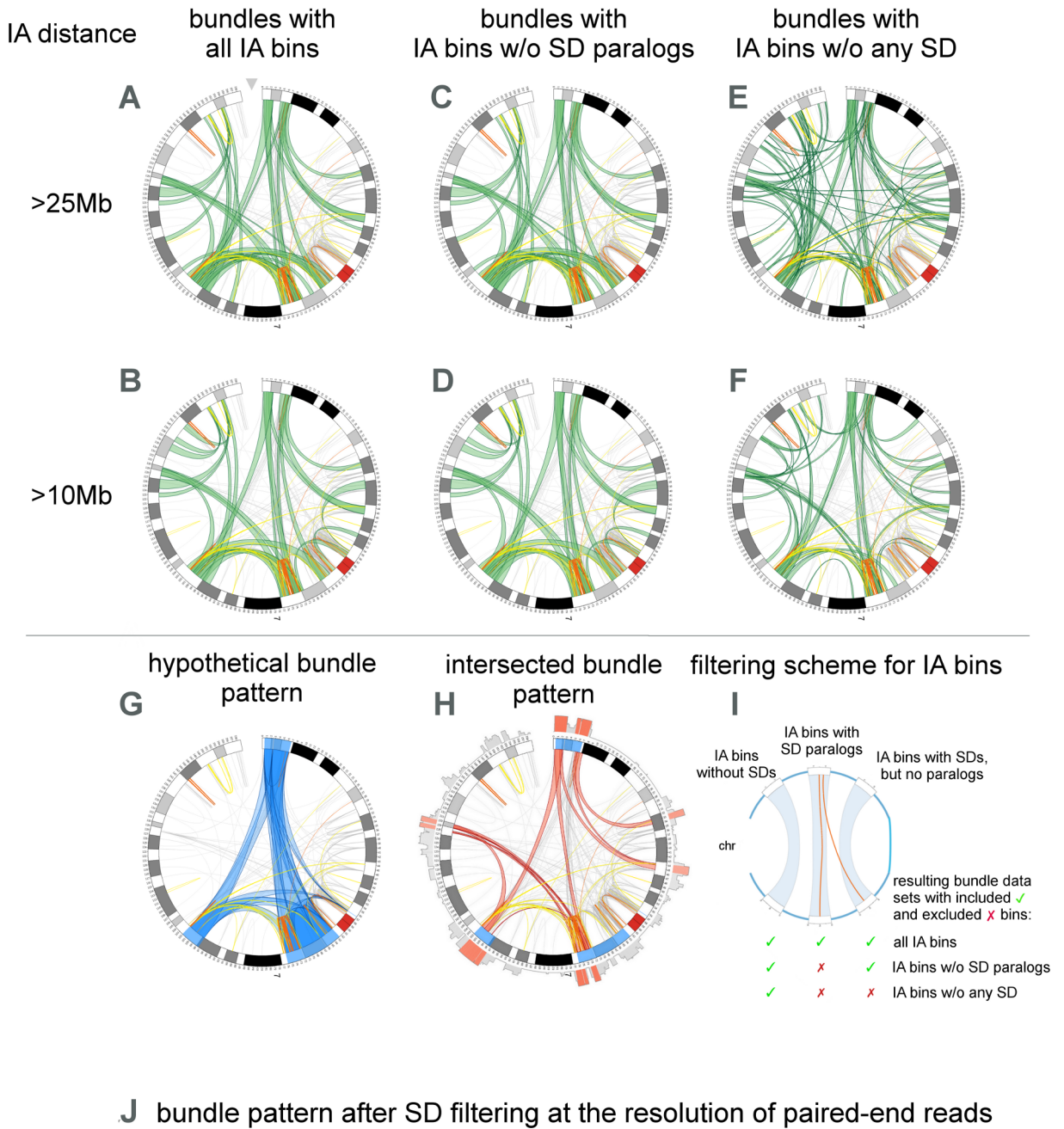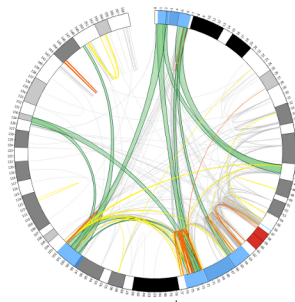

Supplement: Supplementary file 4 — Additional file 4: Changes of long range interaction patterns of human chromosome 7 as a consequence of filtering for interaction counts, span size and SD overlap. Normalised Hi-C interaction data were filtered based on the number of interaction counts (IA) per bin, interaction distance and overlap with SDs. From a total of 12 data sets, only the results for the cut-off “>15 IA/bin” in combination with interaction distance and SD overlap are shown. The first two rows show the effect of using different interaction distances (>25 Mb and >10 Mb) on the interaction bundle pattern (green ribbons). The three columns compare the bundle patterns before and after removing bins covering SDs. The two strategies to exclude bins covering regions with SDs are displayed in I; (w/o - without). all IA bins (A-B): no exclusion of interaction bins based on overlap with SDs; IA bins w/o SD paralogs (C-D): interacting bins were excluded if they connect two paralogous SDs; IA bins w/o any SD (E-F): interaction bins were excluded if they overlap with any SD at all. Visualisation as described in Figure 1. The plot obtained with the filter options used for Figure 1 and Additional file 2 is marked by a grey triangle (A). G) depicts a hypothetical bundle pattern (blue ribbons), which is obtained by connecting all segments of human chromosome 7 that are homologous to marmoset chromosome 2 (highlighted in blue within the idiogram). H) the outer histogram displays the frequency that a given 20 kb bin is part of a bundle region after filtering with the 12 different combinations of cut-offs (red shading within histogram – regions where bins are part of all bundle data sets). Red bundles represent the triangular interaction pattern resulting from bundle regions that are shared by all 12 data sets obtained by various thresholds (see Methods) and additionally map to the same target sites. I) scheme for the removal of bins covered by SDs. J) pattern of long distance interactions after SD filtering at [file 12864_2013_6219_MOESM4_ESM.pdf]

**A**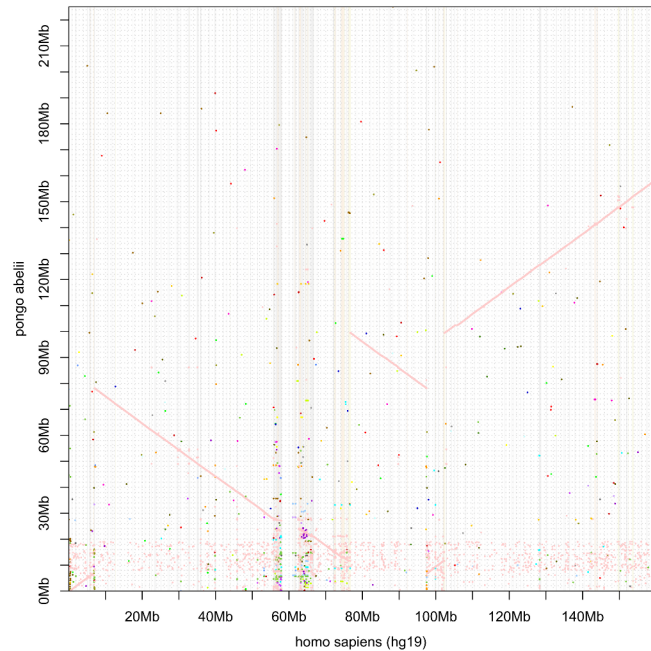**B**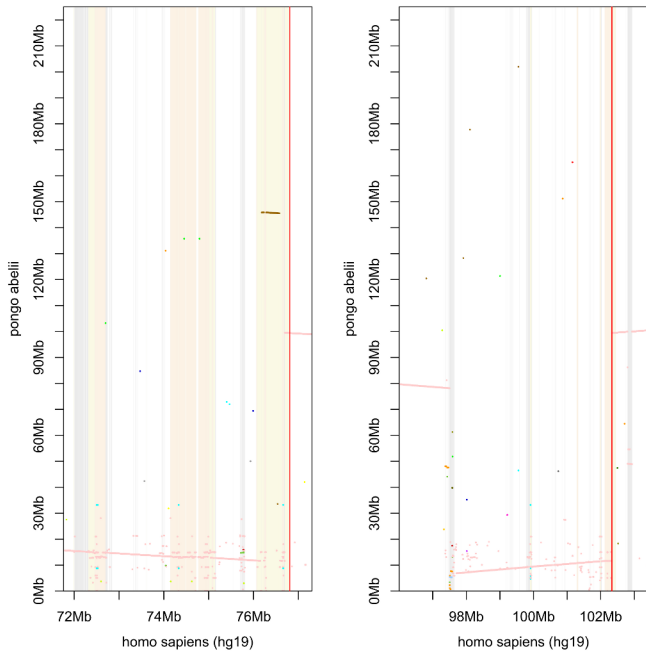

Supplement: Supplementary file 6 — Additional file 6: Dot plots indicating the breakpoints of the paracentric and pericentric inversion. A) Dot plot of human chromosome 7 (hg19) against the orang-utan genome (ponAbe2). B) zoom-in of the proximal and distal breakpoint (vertical red lines) of the paracentric inversion. See text for details. (PDF 650 KB) [file 12864_2013_6219_MOESM6_ESM.pdf]

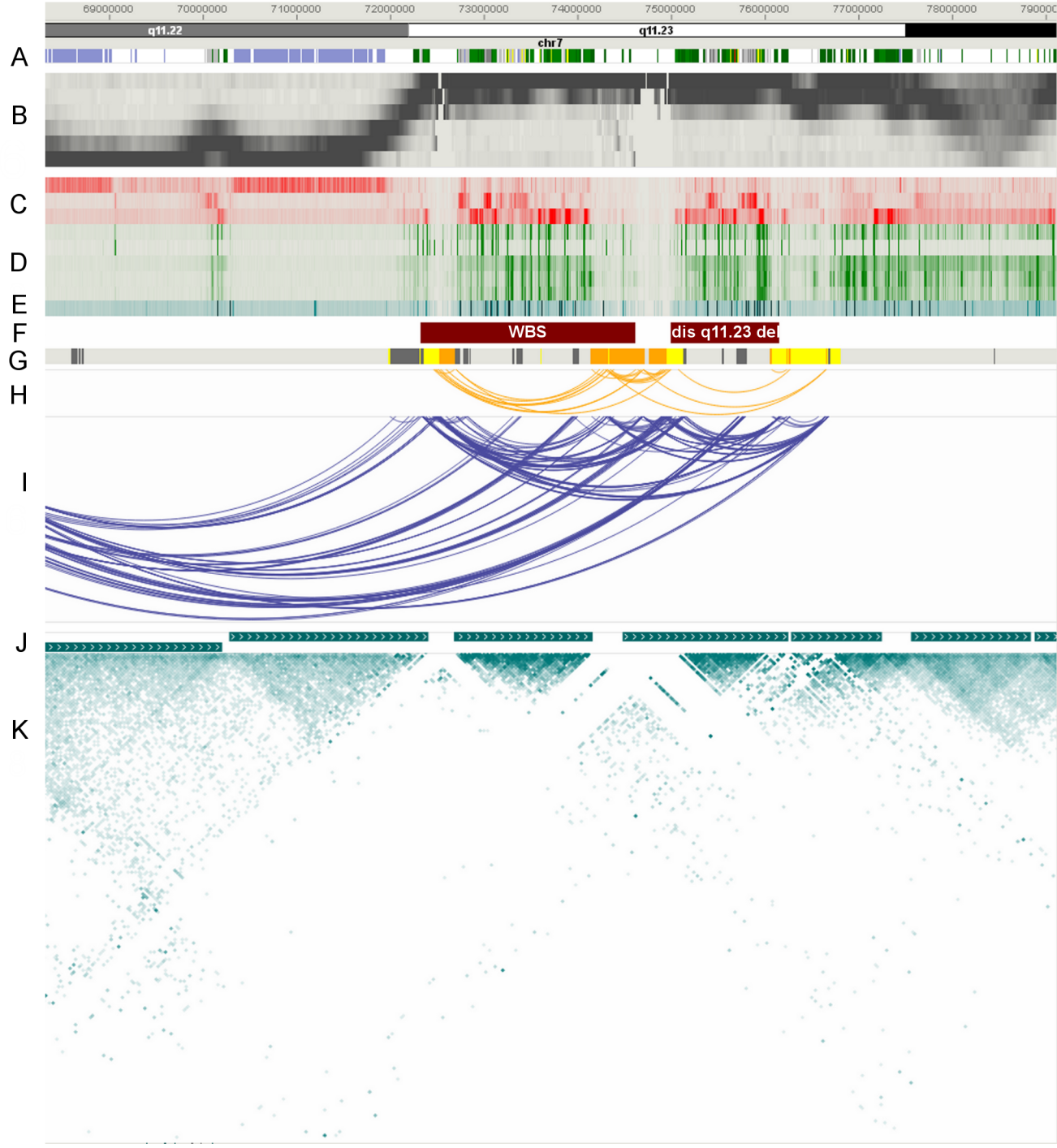

Supplement: Supplementary file 7 — Additional file 7: Additional data on higher order chromatin organisation and SD localisation around the Williams- Beuren syndrome region as depicted in Figure 3 . All data are referring to genome release hg19 and are derived from IMR90. A) chromatin states as defined by [115, 116]; light blue: heterochromatin, dark green: active transcription, yellow: enhancers, grey: weak repression by Polycomb, dark grey: strong repression by Polycomb, red: bivalent poised TSS; B) replication timing phases from G1 (top) to G2 (bottom; [101, 102] in black); C) markers for repressed chromatin (H3K9me3, H3K27me3, H4K20me1) in red; D) markers for active chromatin (H3K4me2, H3K4me3, H3K14ac, H3K18ac, H3K4me1, H3K27ac, H3K36me3) in green; E) CTCF binding sites; F) red bars indicating the intervals typically deleted in WBS and the distal 7q11.23 deletion syndrome; G) distribution of SD blocks following the colouring scheme as described in the Methods section; H) arc view highlighting corresponding SD paralogs with more than 99% sequence similarity; I) arc view highlighting corresponding paralogs for all SDs; J) topological domains defined by Dixon et al. [36]; K) two-dimensional heatmap based on Hi-C data from Dixon et al. [36]. (PDF 2 MB) [file 12864_2013_6219_MOESM7_ESM.pdf]

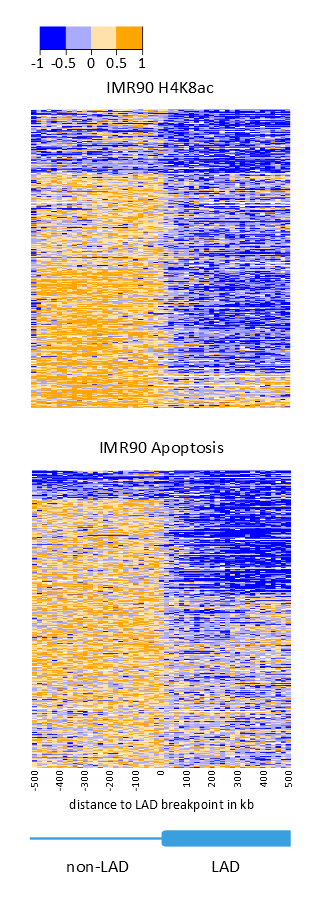

Supplement: Supplementary file 8 — Additional file 8: Comparison of lamina associated domain borders, acetylation of H4K8 and apoptotic DNA degradation. Clustered heatmaps display the degree of H4K8 acetylation and apoptotic DNA degradation 500 kb upstream and downstream of the borders of lamina associated domains (LADs). Data are shown as 20 kb bins. Only LADs with a minimum size of 500 kb and a minimum distance of 500 kb to neighbouring LADs have been selected. DNA degradation and H4K8 acetylation profiles are shown with orange representing genomic regions with a higher rate of apoptotic DNA fragmentation and H4K8 acetylation, while blue depicts less degraded DNA segments and hypoacetylated H4K8, respectively. Visualisation by means of R packages ‘cluster’ [117] and ‘gplots’ [118]. (TIFF 170 KB) [file 12864_2013_6219_MOESM8_ESM.tiff]

A

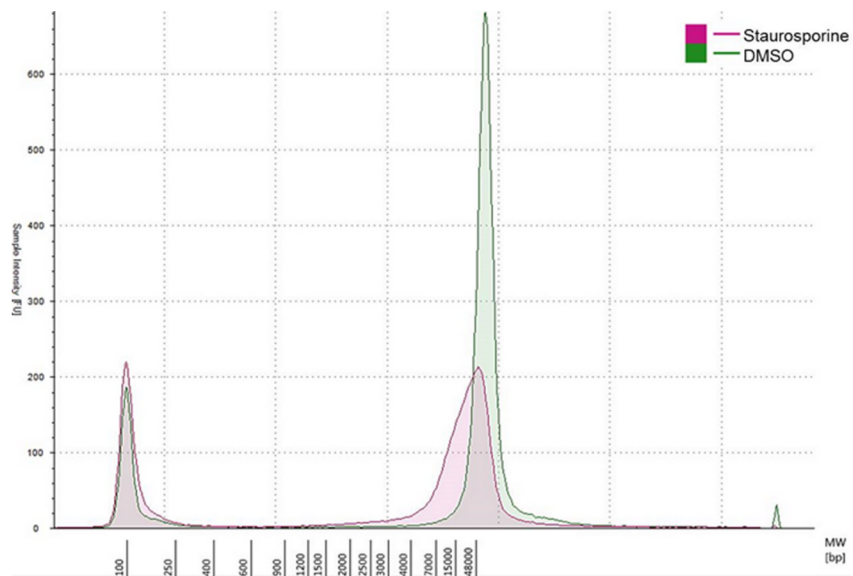

B

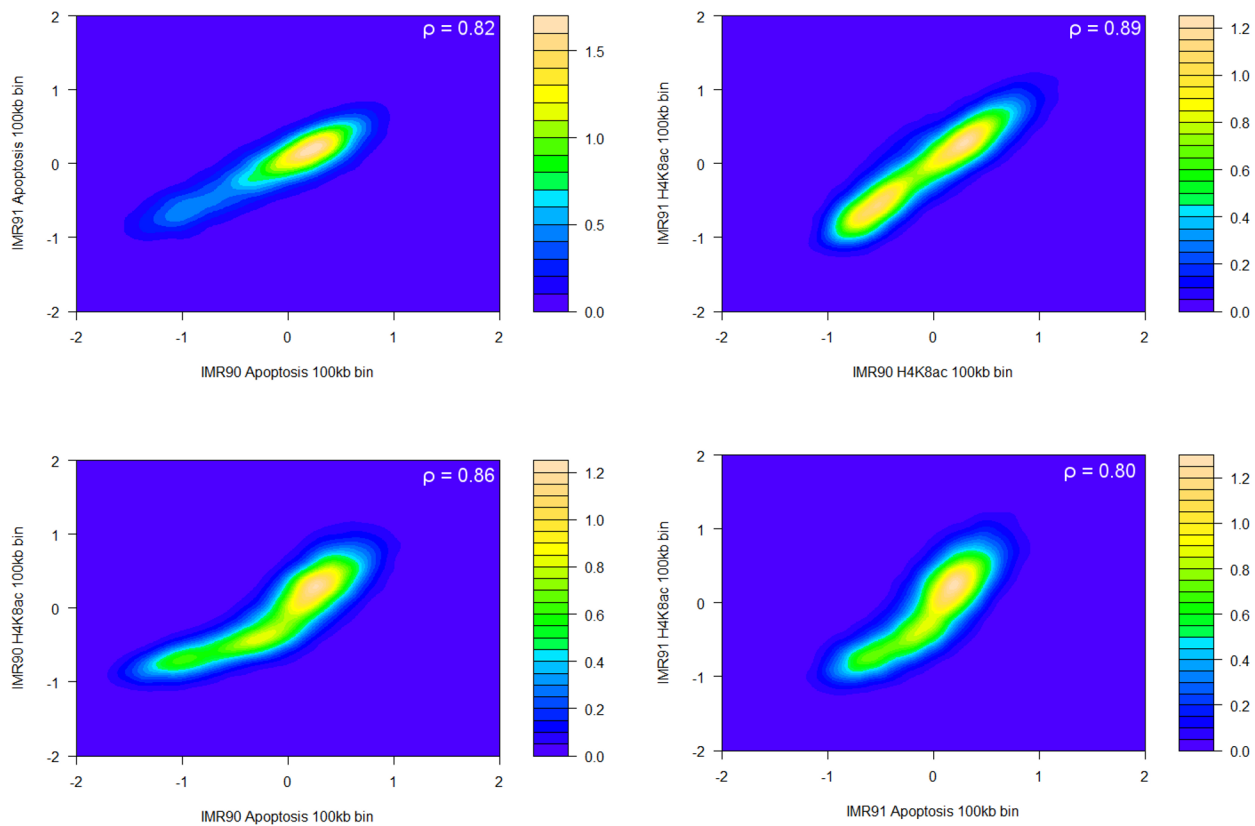

Supplement: Supplementary file 9 — Additional file 9: Reproducibility of apoptotic DNA degradation patterns. A) fragmentation of genomic DNA isolated from IMR90 cells treated with DMSO (control, in green) and after apoptosis induction using staurosporine (in pink) as visualised by an Agilent 2200 Tap2station (Agilent, Santa Clara, USA). B) for visualising the correlation between the IMR90 and IMR91L apoptosis data sets we performed a two-dimensional kernel density estimation ('MASS' R package [113, 119, 120]) of 100 kb binned data under omitting missing values and passed the output to the filled.contour function of the R package 'graphics'. Additionally H4K8 acetylation patterns of the same cell lines were compared; p-value for all plots < 2.2 × 10^−16^. (PDF 822 KB) [file 12864_2013_6219_MOESM9_ESM.pdf]
