# Supplementary material for: Distribution of segmental duplications in the context of higher order chromatin organisation of human chromosome 7
Source: BMC Genomics. 2014 Jun 29;15:537. doi: 10.1186/1471-2164-15-537 (PMC4092221; doi:10.1186/1471-2164-15-537)
Supplement: Supplementary file 10 — Additional file 10: Publicly available data sets used in the study. (DOCX 32 KB) [file 12864_2013_6219_MOESM10_ESM.docx]

| **Data set** | **Reference or GEO accession number** |
| --- | --- |
| Normalized Hi-C interaction data | [[1](#_ENREF_1)] |
| Segmental duplications | [[2](#_ENREF_2), [3](#_ENREF_3)] |
| Alu repeats | [[4](#_ENREF_4)] |
| G4 DNA motifs | [[5](#_ENREF_5)] |
| lamin B1 interaction sites | [[6](#_ENREF_6)] |
| lamina-associated domains (LADs) | [[6](#_ENREF_6)] |
| replication timing | [[7](#_ENREF_7), [8](#_ENREF_8)] |
| CTCF ChIP-seq signals | GSM935404 |
| CTCF ChIA-PET interactions (MCF-7) | [[9](#_ENREF_9)], GSM970215 |
| topological domains | [[1](#_ENREF_1)] |
| Database of Genomic Variance (DGV) | [[10](#_ENREF_10)] |
| genomic disorders (DECIPHER) | [[11](#_ENREF_11)] |
| posttranslational histone modifications | Additional file 9 |

**Additional file 10:** **Publicly available data sets used in the study**

1. Dixon JR, Selvaraj S, Yue F, Kim A, Li Y, Shen Y, Hu M, Liu JS, Ren B: **Topological domains in mammalian genomes identified by analysis of chromatin interactions.** *Nature* 2012, **485:**376-380.

2. Bailey JA, Gu ZP, Clark RA, Reinert K, Samonte RV, Schwartz S, Adams MD, Myers EW, Li PW, Eichler EE: **Recent segmental duplications in the human genome.** *Science* 2002, **297:**1003-1007.

3. Bailey JA, Yavor AM, Massa HF, Trask BJ, Eichler EE: **Segmental duplications: Organization and impact within the current Human Genome Project assembly.** *Genome Research* 2001, **11:**1005-1017.

4. **RepeatMasker Open-3.0** [<http://www.repeatmasker.org/>]

5. Cer RZ, Donohue DE, Mudunuri US, Temiz NA, Loss MA, Starner NJ, Halusa GN, Volfovsky N, Yi M, Luke BT, et al: **Non-B DB v2.0: a database of predicted non-B DNA-forming motifs and its associated tools.** *Nucleic acids research* 2013, **41:**D94-D100.

6. Guelen L, Pagie L, Brasset E, Meuleman W, Faza MB, Talhout W, Eussen BH, de Klein A, Wessels L, de Laat W, van Steensel B: **Domain organization of human chromosomes revealed by mapping of nuclear lamina interactions.** *Nature* 2008, **453:**948-951.

7. Hansen RS, Thomas S, Sandstrom R, Canfield TK, Thurman RE, Weaver M, Dorschner MO, Gartler SM, Stamatoyannopoulos JA: **Sequencing newly replicated DNA reveals widespread plasticity in human replication timing.** *Proc Natl Acad Sci U S A* 2010, **107:**139-144.

8. Thurman RE, Day N, Noble WS, Stamatoyannopoulos JA: **Identification of higher-order functional domains in the human ENCODE regions.** *Genome Res* 2007, **17:**917-927.

9. Li G, Fullwood MJ, Xu H, Mulawadi FH, Velkov S, Vega V, Ariyaratne PN, Mohamed YB, Ooi HS, Tennakoon C, et al: **ChIA-PET tool for comprehensive chromatin interaction analysis with paired-end tag sequencing.** *Genome Biol* 2010, **11:**R22.

10. Iafrate AJ, Feuk L, Rivera MN, Listewnik ML, Donahoe PK, Qi Y, Scherer SW, Lee C: **Detection of large-scale variation in the human genome.** *Nature genetics* 2004, **36:**949-951.

11. Firth HV, Richards SM, Bevan AP, Clayton S, Corpas M, Rajan D, Van Vooren S, Moreau Y, Pettett RM, Carter NP: **DECIPHER: Database of Chromosomal Imbalance and Phenotype in Humans Using Ensembl Resources.** *American journal of human genetics* 2009, **84:**524-533.
